# Spatially and Temporally Confined Response of Gastrointestinal Antibiotic Resistance Gene Levels to Sulfadiazine and Extracellular Antibiotic Resistance Gene Exposure in Mice

**DOI:** 10.3390/biology12020210

**Published:** 2023-01-29

**Authors:** Xin Wei, Jian Zhang, Bianfang Wang, Wenjia Wang, Yuqing Sun, Ling Li, Hai Xu, Mingyu Wang

**Affiliations:** State Key Laboratory of Microbial Technology, School of Life Sciences, Shandong University, Qingdao 266237, China

**Keywords:** eARGs, gastrointestinal tract, mouse model, antimicrobial resistance, spatiotemporal distribution, sulfadiazine

## Abstract

**Simple Summary:**

The emergence of bacterial resistance to antibiotics is a significant threat to human health, because it makes controlling bacterial infection more difficult. Antibiotic resistance genes that can cause bacteria to resist antibiotics are ubiquitous in the environment and consumed daily by humans via food and water. It has long been suspected that uptake of these genes may lead to increased antibiotic resistance in bacteria that colonize human bodies, which in turn may lead to infections that are hard to cure. In addition, the uptake of antibiotics may also lead to an increase in antibiotic resistance, resulting in negative impacts for further treatment. To address these concerns, we applied an in vivo study to determine how the antibiotic resistance levels in the gastrointestinal tracts of mice models react to uptake of antibiotics and antibiotic resistance genes. With quantitative analysis, both antibiotics and antibiotic resistance genes were found to indeed increase antibiotic resistance levels. However, this response was found to be both temporally and spatially confined: they are acute responses that only occur 12–16 days after exposure, and they only occur in certain segments of the gastrointestinal tract. This work further suggests caution over antibiotic resistance gene pollution and antibiotic misuse.

**Abstract:**

This work aims to investigate the impact of antibiotics and extracellular antibiotic resistance genes (eARGs) on the dynamics of gastrointestinal antimicrobial resistance (AMR). The antibiotic resistance gene (ARG) levels of different segments of the gastrointestinal tract of mouse models were analyzed and compared after exposure to clinical concentrations of sulfadiazine and environmental levels of eARGs carried by the conjugative plasmid pR55. Exposure to sulfadiazine and eARGs led to significant changes in ARG levels by as many as four log-folds. Further analysis showed that the response of ARG levels appeared from 12–16 days after exposure and diminished 20 days after exposure. The responses in ARG levels were also restricted to different gastrointestinal segments for sulfadiazine and eARGs. Combined exposure of sulfadiazine and eARGs was unable to further increase ARG levels. From these findings, we concluded that the short-term consumption of environmental levels of eARGs and uptake of clinical levels of antibiotics lead to a spatially and temporally confined response in gastrointestinal AMR. These findings further clarify the detrimental impacts of antibiotic and eARG uptake, and the complexity of AMR development and dissemination dynamics in the gastrointestinal tract.

## 1. Introduction

Antimicrobial resistance (AMR) has been deemed an imminent threat to mankind due to its reduction of antibiotic efficacies. Antibiotic resistance genes (ARGs) that can be hosted by various pathogens and can be transmitted between cells could be dangerous once they enter human pathogens, including opportunistic pathogens naturally present in gastrointestinal microbiomes [1]. Transfer of ARGs from the environment to human gut microbiota has been observed [2]. Furthermore, high levels of ARGs may be transferred to other body parts, aggravating bacterial infections taking place elsewhere [3]. Therefore, ARGs are widely considered a detrimental pollution potentially affecting human health.

Antibiotic resistance genes are present both intracellularly (intracellular ARGs, iARGs) and extracellularly (extracellular ARGs, eARGs) in the environment [4]. ARGs are present at low concentrations in nearly every aspect of the biosphere including air, water and soil, and have been extensively examined in the environment for their potential threats [5,6]. Due to the low efficiencies of water treatment plants in removing ARGs, even tap water contains high levels of ARGs, making them a common pollutant [7]. Food was also found to contain a low, yet significant, amount of ARGs acquired from soil [8]. Therefore, eARGs are constantly consumed by humans, which, in theory, might increase the overall level of ARGs in gastrointestinal bacteria, further leading to an increase in intestinal pathogens’ resistance to antibiotic treatment, potentially leading to high risks of infection. However, little has been done experimentally to determine whether the low levels of eARGs consumed via food and drinking water actually have any substantial impacts.

Plenty of work has been done on AMR and its transferability in intestinal microbiomes both in vivo and in vitro. Antibiotics are consumed both orally or intravenously to combat bacterial infections, and substantial work has been carried out on the impact of intestinal AMR by antibiotics. Antibiotic treatment significantly decreases the diversity of intestinal microflora, promotes AMR in the gastrointestinal tract, and leads to dysbiosis [9]. ARG transferability has been investigated at both the single organism and microbiome level to find out whether ARGs carried by non-pathogenic bacteria may be transferred to pathogens, making their infections harder to cure [10]. The metagenomic and resistomic aspects of intestinal microbiomes have been investigated in detail, revealing a complex network [11]. The AMR at different intestinal segments was also investigated in animal models including mice, pigs, and zebrafishes [12,13,14]. Nevertheless, the spatiotemporal distribution and dynamics of ARGs in gastrointestinal tract in response to antibiotics remain to be studied.

In this work, we aim to expand our understanding of the AMR of gastrointestinal microbiomes by examining the consequences of eARG uptake and antibiotic uptake. In particular, using sulfadiazine as a representative antibiotic and eARGs in the form of a conjugative multidrug-resistant plasmid, we aim to determine whether environmental levels of eARGs can lead to increased AMR levels in the gastrointestinal tract of mouse models, the response of AMR in different gastrointestinal segments to antibiotics and eARGs, and how a combination of antibiotics and eARGs may impact gastrointestinal AMR levels. Finding out answers to these questions helps to unravel the influences of clinical antibiotics and environmental eARG pollution on human health, and also the impact of environmental dissemination of ARGs and clinical antibiotic uptake.

## 2. Materials and Methods

### 2.1. Plasmids and Strains

The conjugative multidrug-resistant plasmid pR55 of the IncA/C group (Genbank accession No. JQ010984.1) is a kind gift from Prof. Hongning Wang from Sichuan University [15]. This *Klebsiella pneumoniae*-hosted plasmid is a 171 kbp sized plasmid that harbors the ARGs *floR*, *sul1*, *sul2*, *catI*, *bla*_OXA-21_, *qacEΔ1*, and *aadB*. The pR55 plasmid also contains a type I integron that carries a multidrug *aadB*-*bla*_OXA-2_ gene cassette [15]. Transmission of pR55 between *K. pneumoniae* and other *Enterobacteriaceae* is frequently observed. The *Escherichia coli* strain C600 containing pR55 was used as the donor for conjugation. The *Salmonella enterica* strain H9812 was used as the recipient for conjugation.

### 2.2. Conjugation Assay

Conjugation of pR55 between *E. coli* C600 and *S. enterica* H9812 was performed following previously published protocols [16,17]. The *Salmonella*-selective SS medium (*Salmonella*-*Shigella* Agar, Hope Bio-Technology co., ltd, Qingdao, China) was used to screen for the presence of transconjugants. Chloramphenicol was added to the media to screen for pR55-harboring *S. enterica* H9812.

### 2.3. Exposure of Gastrointestinal Microbiomes to Sulfadiazine, pR55, and Sulfadiazine + pR55

**Figure 1:**
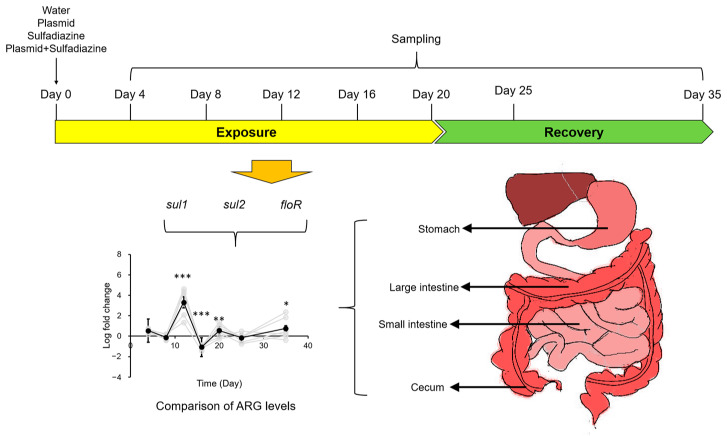
Experimental design of this study. *, *p* < 0.05; **, *p* < 0.01; ***, *p* < 0.001.

To answer the question of how antibiotics and eARGs influence AMR in mouse gastrointestinal microbiomes, four experiments were designed: (1) mice were fed with water (the negative control); (2) mice were fed with clinical dosages of sulfadiazine; (3) mice were fed with environmental levels of eARGs in the form of the conjugative multidrug-resistant plasmid pR55; and (4) mice were fed with both clinical dosages of sulfadiazine and environmental-level pR55. A total of 168 9-week-old Specific Pathogen Free (SPF) grade C57BL/6 mice (84 males, 84 females) were purchased from Beijing Vital River Laboratory Animal Technology Co., Ltd. and raised in individual ventilated cages (Shanghai Tianhuan Technologies co., Ltd.), with the temperature at 22 ± 2 °C, relative humidity at 50 ± 5%, and a luminescence cycle of 12 h/12 h (light/dark). Sterilized water and feed prepared with an autoclave (121 °C, 20 min) were fed to mice to avoid external microbial contamination. The mice were fed with water, sulfadiazine (5.76 mg day^−1^), pR55 solution (1.5 × 10^7^ copies∙day^−1^), and a combination of sulfadiazine + pR55 by gavage daily for 20 days, followed by 15 days of recovery. Sulfadiazine was used as the investigated antibiotic because pR55 carries resistance genes *sul1* and *sul2* for this antibiotic, making investigation of the interaction between antibiotic exposure and eARG exposure more relevant. Mice were grouped into six groups (3 male and 3 female groups). One mouse in each group was sacrificed every 4 days during exposure and twice (at Day 25 and Day 35) during recovery. Different segments of the gastrointestinal tract (stomach, small intestine, cecum and large intestine) were dissected, and the contents were extracted for analysis of ARG levels. An illustration of the experimental setup is shown in Figure 1.

Total DNA was extracted from the samples using the guanidine thiocyanate (GITC) method for further quantification of ARGs [18]. Specifically, for sample pretreatment, 50–100 mg samples were mixed with 50–100 mg quartz sand and 900 μL Phosphate Buffered Saline (PBS, 0.1 M, pH 8.0), vortexed for 5 min, and centrifuged for 5 min at 13,000 rpm. The precipitate was washed three times before the next round of pretreatment. Three pretreatment cycles were performed. Pretreated samples were dissolved in 800 μL PBS containing 20 μL lysozyme (50 mg mL^−1^), and heated in a water bath at 37 °C for 1 h. Fifty microliters of Sodium Dodecyl Sulfate (SDS) mixture (0.25 M NaCl, 0.1 M EDTA, 20% SDS) and 100 μL GITC (5 M) were subsequently added to the mixture and heated at 65 °C for 30 min. GITC-treated samples were centrifuged at 10,000×*g* rpm for 10 min. The supernatant was supplemented with potassium acetate (0.125 volume, 5 M) and Polyethylene Glycol (PEG) 8000 (0.42 volume, 40%), and placed in an ice bath for 15 min. The solution was further centrifuged at 14,000×*g* rpm for 10 min. The precipitation was mixed gently with 700 μL Tris-EDTA (TE: 10 mM Tris-HCl, 1 mM EDTA, pH 8.0) and 700 μL chloroform:isopentanol solution (24:1), and centrifuged at 12,000×*g* rpm for 5 min. The supernatant was incubated with pre-cooled ethanol and placed in an ice bath for 1–2 h, followed by centrifugation at 12,000 rpm for 10 min. The precipitate was washed with 70% ethanol, dried, and dissolved in 50 μL sterile water for further analysis.

### 2.4. Quantitative Methods

Quantification of ARGs was performed by qPCR with a Real-Time PCR system (Type StepOnePlus, Applied Biosystems, Waltham, MA, USA) using SYBR Premix Ex Taq II (Tli RNaseH Plus, Takara Bio Inc., Shiga, Japan), with similar protocols to reported procedures [19]. Standard curves were prepared with the gene-harboring pMD19-T vector. Three technical replicates were performed for each reaction. The primers used for quantification and other parameters are shown in Table S1 [20,21,22]. The melting curves for the reactions were previously reported [20] or shown in Figure S1 (for *sul2*).

### 2.5. Statistics

The comparison of ARG levels (in copies∙mg^−1^) was carried out with the two-tailed Mann–Whitney U test using the Jamovi v2.3.21 software. For comparison and data plotting, changes of log ARG levels *versus* the water control were calculated. A value of *p* < 0.05 was considered statistically significant.

## 3. Results and Discussion

### 3.1. Experimental Setup

The experimental setup as detailed in Section 2.3 allows us to investigate how exposure to antibiotics, eARGs, and a combination of both influence ARG levels in the gastrointestinal tract of mouse models. The conjugative experiment was performed to ensure the transferability of the eARG-carrying pR55 by showing the transfer of pR55 from *E. coli* C600 to *S. enterica* H9812 (Figure S2). This experimental design mimics real world scenarios. The amount of sulfadiazine used in exposure corresponds to the daily dosage used in adult humans (2000 mg · 60 kg^−1^ day^−1^). This dosage was used to mimic oral sulfadiazine uptake for infection prevention and cure. The levels of conjugative pR55 plasmid used in exposure in this experiment mimics the levels of ARGs consumed by mice if they had been exposed to environmental levels of eARGs (1.5 × 10^7^ copies *sul2*∙day^−1^) [23]. The uptake of both sulfadiazine and plasmid mimics the scenario where individuals take antibiotics while consuming eARG-contaminated water. It needs to be explained here that naked plasmids rather than plasmid-containing microbes were used because microbe-hosted plasmids would have been considered iARGs, thereby defeating the purpose of this study. Conjugative plasmids were used because they are expected to have a higher probability of entering bacterial cells for further amplification and transfer than simple DNA fragments, therefore making potential AMR changes in gastrointestinal tract more visible. It also needs to be noted that in this experimental design, three ARGs in pR55 were followed. This does not mean that these ARGs are not naturally present in the gastrointestinal tract microbiomes of mice. Despite their presence in the background, an increased dissemination of ARGs will still lead to a surge in ARG levels above the background level, representing an elevation of overall AMR levels.

### 3.2. Impact of Sulfadiazine and eARG Exposure on ARG Levels in Gastrointestinal Tract of Mice

**Figure 2:**
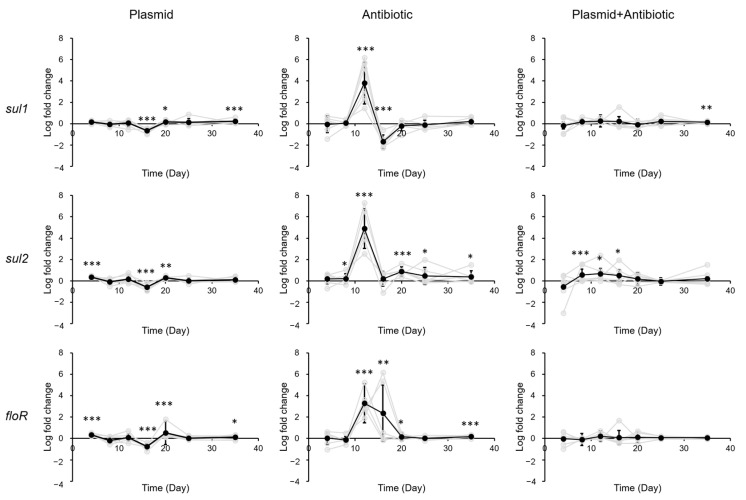
Change of ARG levels in mouse stomach after exposure to pR55 (plasmid), sulfadiazine (antibiotic), or pR55 + sulfadiazine (plasmid + antibiotic). Each gray line indicates a mouse group (male groups 1–3, female groups 1–3). Black line indicates average of all six groups. Error bars indicate standard deviation of 6 replicates (3 males and 3 females). *, *p* < 0.05; **, *p* < 0.01; ***, *p* < 0.001.

Analysis of ARG levels in gastrointestinal tract of mice after exposure to sulfadiazine, eARGs and sulfadiazine + eARGs led to the observation of different patterns in different segments of the gastrointestinal tract. In the stomach, exposure of pR55 or sulfadiazine + pR55 led to very little changes in ARG levels (Figure 2), whereas sulfadiazine exposure alone led to a strong (2–6 log, average 3.99 log) and significant increase of ARG levels 12 days after exposure, which quickly returned to pre-exposure levels in 4–8 days.

**Figure 3:**
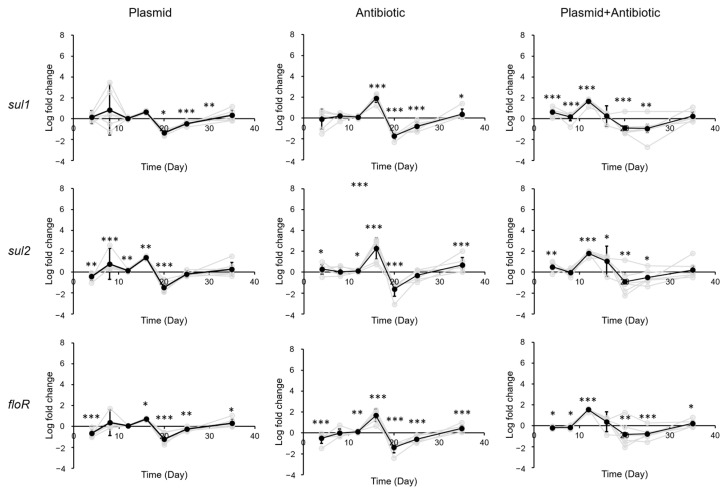
Change in ARG levels in mouse small intestine after exposure to pR55 (plasmid), sulfadiazine (antibiotic), or pR55 + sulfadiazine (plasmid + antibiotic). Each gray line indicates a mouse group (male groups 1–3, female groups 1–3). Black line indicates average of all six groups. Error bars indicate standard deviation of 6 replicates (3 males and 3 females). *, *p* < 0.05; **, *p* < 0.01; ***, *p* < 0.001.

In the small intestine, exposure to eARGs also led to only minimal changes in ARG levels. In comparison to the stomach, a smaller (1–3 log, average 1.95 log) yet still significant increase in ARG levels can be found in the small intestine 16 days after exposure to antibiotic (Figure 3). A similar increase can be found after exposure to plasmid + antibiotic, except that the response took place 4 days earlier than for exposure to antibiotic. Similar to the stomach, this increase diminished towards the end of the exposure (20 days after exposure).

**Figure 4:**
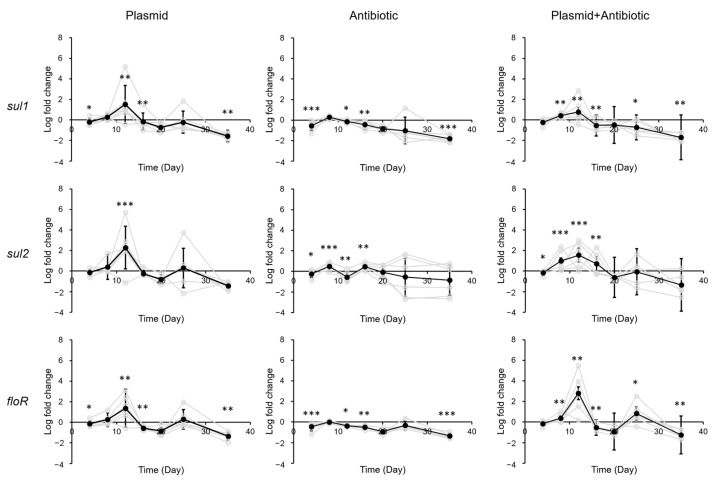
Change in ARG levels in mouse cecum after exposure to pR55 (plasmid), sulfadiazine (antibiotic), or pR55 + sulfadiazine (plasmid + antibiotic). Each gray line indicates a mouse group (male groups 1–3, female groups 1–3). Black line indicates average of all six groups. Error bars indicate standard deviation of 6 replicates (3 males and 3 females). *, *p* < 0.05; **, *p* < 0.01; ***, *p* < 0.001.

In the cecum, contrary to what was observed in the stomach, exposure to plasmid and plasmid + antibiotic led to a significant increase in ARG levels (1–3 log, average 1.71 log) 12 days after exposure, whereas exposure to antibiotic led to little difference (Figure 4). This increase also disappeared after 4–8 days.

**Figure 5:**
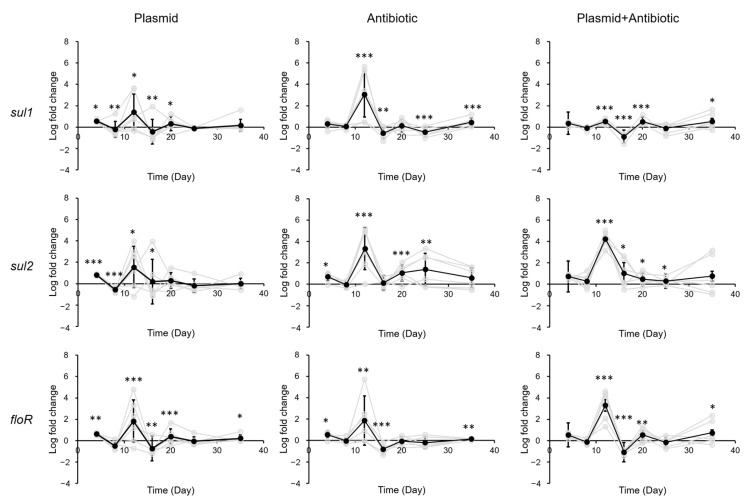
Change in ARG levels in mouse large intestine after exposure to pR55 (plasmid), sulfadiazine (antibiotic), or pR55 + sulfadiazine (plasmid + antibiotic). Each gray line indicates a mouse group (male groups 1–3, female groups 1–3). Black line indicates average of all six groups. Error bars indicate standard deviation of 6 replicates (3 males and 3 females). *, *p* < 0.05; **, *p* < 0.01; ***, *p* < 0.001.

In the large intestine, exposure to either plasmid, antibiotic, or plasmid + antibiotic led to increased ARG levels (plasmid, average 1.59 log; antibiotic, average 2.76 log; plasmid + antibiotic, average 2.69-fold) 12 days after exposure (Figure 5). No significant difference was found between the three conditions using two-tailed pairwise *t*-tests or Mann–Whitney U tests. This increase, similar to that in the other gastrointestinal tract segments, was no longer seen four days later.

A common and significant phenomenon in each segment of the gastrointestinal tract we observed is that the increase of ARGs following exposure to antibiotic, plasmid, or plasmid and antibiotic combined is temporally confined. It only took place 12–16 days after exposure and disappeared 20 days after exposure. This common phenomenon leads us to believe this observed surge of AMR in gastrointestinal tract is an acute response that may not carry long-term consequences, which can be defined with further long-term exposure experiments. This is of particular significance for the case of eARGs, as eARGs are prevalent in the biosphere and routinely consumed as contaminants in food and water.

In addition to the apparent and significant temporally confined patterns of ARG response, it is evident that the response is also spatially confined: each segment of the gastrointestinal tract has a different response pattern. The eARGs were unable to stimulate ARG levels in the stomach, and antibiotics were unable to stimulate ARG levels in the cecum. A combination of eARGs and antibiotics was unable to stimulate ARG levels in the stomach, which is surprising since sulfadiazine alone can stimulate ARG levels. Generally, higher levels of stimulation were found for sulfadiazine than pR55 (*p* = 0.039, one-tailed Mann–Whitney test, null hypothesis: sulfadiazine does not stimulate ARG levels more than pR55).

It has long been known that antibiotics can promote both horizontal and vertical gene transfer of ARGs [24]. Both in vitro and in vivo experiments showed transfer of ARGs to gastrointestinal microbes under the pressure of antibiotics [9,16], although most of these investigations focused on the transferability rather than the association of overall AMR levels to antibiotic pressure. ARG-carrying bacteria are better capable of dealing with the bactericidal effects of antibiotics. Therefore, in the presence of antibiotics, they out-perform bacteria that do not carry ARGs. This gives them a selective advantage which leads to a higher proportion of ARG-carrying bacteria, which in turn leads to higher overall ARG levels. However, antibiotic treatment may result in metabolic adaptation without changes in genetic content, leading to higher resistance to antibiotics [25]. When the selective disadvantage in non-ARG-carrying bacteria is weakened by metabolic adaptation to a point where it is comparable to the fitness cost of synthesizing plasmids in ARG-carrying bacteria, ARG levels may cease to be stimulated by antibiotics.

Few investigations attempted to answer the question of how eARGs, particularly at environmental levels, impact gastrointestinal AMR. Plasmids carrying eARGs can enter bacterial cells by natural transformation. Plasmid-carrying bacteria can duplicate these plasmids, leading to increased ARG levels. This is suspected to be the primary reason for the stimulation of ARG levels by plasmids. However, these plasmids are prone to be lost without evolutionary stress to maintain them. In addition, plasmids may pose fitness costs to bacteria [26], leading to weakened competition with other microbes. Theoretically, the presence of antibiotics elicits selective pressure for better maintenance of ARG-carrying plasmids and may lead to the prolonged increase of ARG levels. However, this effect was not observed, agreeing with the above hypothesis that the selective advantage of ARG-carrying microbes under antibiotic pressure diminishes as metabolic adaptation of ARG non-carrying bacteria increases tolerance to antibiotics towards the end of the exposure.

With these considerations, the increase in ARG levels in the first 16 days of exposure to plasmids and antibiotics can be explained by the initial selective advantage of ARG-carrying microbes in the presence of antibiotics, and the vertical transfer and duplication of ARGs in the presence of plasmids. The following drop can be explained by the lack of selective advantage to maintain the plasmids, or metabolic adaption-led reduced selective advantage that is eventually offset by the fitness costs of carrying large plasmids. This automated correction mechanism reflects the resilience of natural gastrointestinal microbiomes to external turbulences.

The different patterns of ARG increase in different gastrointestinal tract segments can be attributed to the different characteristics of the environment and the microbiomes in each segment. In the stomach, the microbiomes are, in general, quite simple, and may not contain proper hosts for pR55 [27]. Therefore, pR55 gavage was unable to increase ARG levels. In the small intestine, the increase in ARGs is smaller than in other segments, which can be attributed to the faster turnover of microbiomes [28] and subsequently lower vertical gene transfer and competition in the small intestine, given that bacterial levels in large intestine are 7–8 orders of magnitude higher than in the small intestine [29]. In the cecum, antibiotics were unable to stimulate ARG levels, and the stimulation of ARGs was weak, which may be attributed to the lack of stimulation of antibiotics in the cecum, as the movement of intestinal contents partially bypasses this segment. The response of ARGs is most pronounced in the large intestine, as it is the intestinal segment that is the richest in its microbiome, where horizontal gene transfer, competition, and all other microbiota-related processes are the most active.

Results obtained in this work confirm that both environmental level eARGs in the form of conjugative plasmids and antibiotics can promote AMR in gastrointestinal tracts using mouse models. These findings confirm that both taking antibiotics and consuming eARG-polluted food or drink can reduce the efficacy of further antibiotic treatments. Taking antibiotics while consuming eARGs was not found to further improve AMR in the gastrointestinal tract. However, the detrimental impacts of eARGs and antibiotics were found to be temporary, and represents acute responses, whereas putative long-term impacts were not investigated. These impacts were also found to be localized in specific segments along the gastrointestinal tract, suggesting that these impacts are not universal, although in the large intestine where microbial activities and interactions are the most robust, both eARGs and antibiotics can stimulate ARG levels. These results reveal in vivo AMR responses of consuming antibiotics and eARGs, and lead to a better understanding of the AMR development kinetics in the gastrointestinal tract where the largest microbial community in human bodies are present.

## 4. Conclusions

The impact of antibiotics and eARGs on AMR in the gastrointestinal tract was investigated in this work with mouse models. Both environmental levels of eARGs, in the form of conjugative plasmid pR55, and clinical levels of antibiotics, represented by sulfadiazine, are capable to stimulating an ARG surge in the gastrointestinal tract, confirming their detrimental impacts. However, the stimulation by both eARGs and antibiotics are temporally and spatially confined: ARG levels in gastrointestinal tracts return to baseline 20 days after exposure, and these responses are absent in several segments of the gastrointestinal tract. Addition of antibiotics to eARGs was unable to further stimulate ARG levels. These findings provide evidence that supports the cautious application of antibiotics and the necessity of removing eARGs from the environment.

## Data Availability

The data presented in this study are available in this article and Supplementary Materials.

## Supplementary Materials

The following supporting information can be downloaded at: https://www.mdpi.com/article/10.3390/biology12020210/s1, Table S1: Primers used in qPCR reactions; Figure S1: Melting curve for primers targeting *sul2*; Figure S2: Conjugative transfer of pR55 from *E. coli* C600 to *S. enterica* H9812.
