# Supplementary material for: Spatially and Temporally Confined Response of Gastrointestinal Antibiotic Resistance Gene Levels to Sulfadiazine and Extracellular Antibiotic Resistance Gene Exposure in Mice
Source: Biology (Basel). 2023 Jan 29;12(2):210. doi: 10.3390/biology12020210 (PMC9953105; doi:10.3390/biology12020210)

Supplementary Materials

Table S1. Primers used in qPCR reactions.

Target gene	Primer sequence (5´-3´)	Product size (bp)	Quantification formula	R^2	Amplification efficiency
sul1	F: CGCACCGGAAACATCGCTGCAC<br>R: TGAAGTTCCGCCGCAAGGCTCG	162	Copy number $=9\times10^{11-0.711Cq}$	0.9947	1.04
sul2	F: TCATCTGCCAAACTCGTCGTTA<br>R: GTCAAAGAACGCCGCAATGT	105	Copy number $=2\times10^{12-0.734Cq}$	0.9977	1.08
floR	F: GAGGGTGTCGTCATCTACGG<br>R: GAGCATCGCCAGTATAGCCA	138	Copy number $=1\times10^{12-0.648Cq}$	0.9991	0.91
16S rDNA	F: CCCAGATGGGATTAGCTTGT<br>R: TCTGGACCGTGTCTCAGTTC	106	Copy number $=2\times10^{16-0.722Cq}$	0.9907	1.07

Figure S1. Melting curve for primers targeting *sul2*. Different curves represent technical replicates.

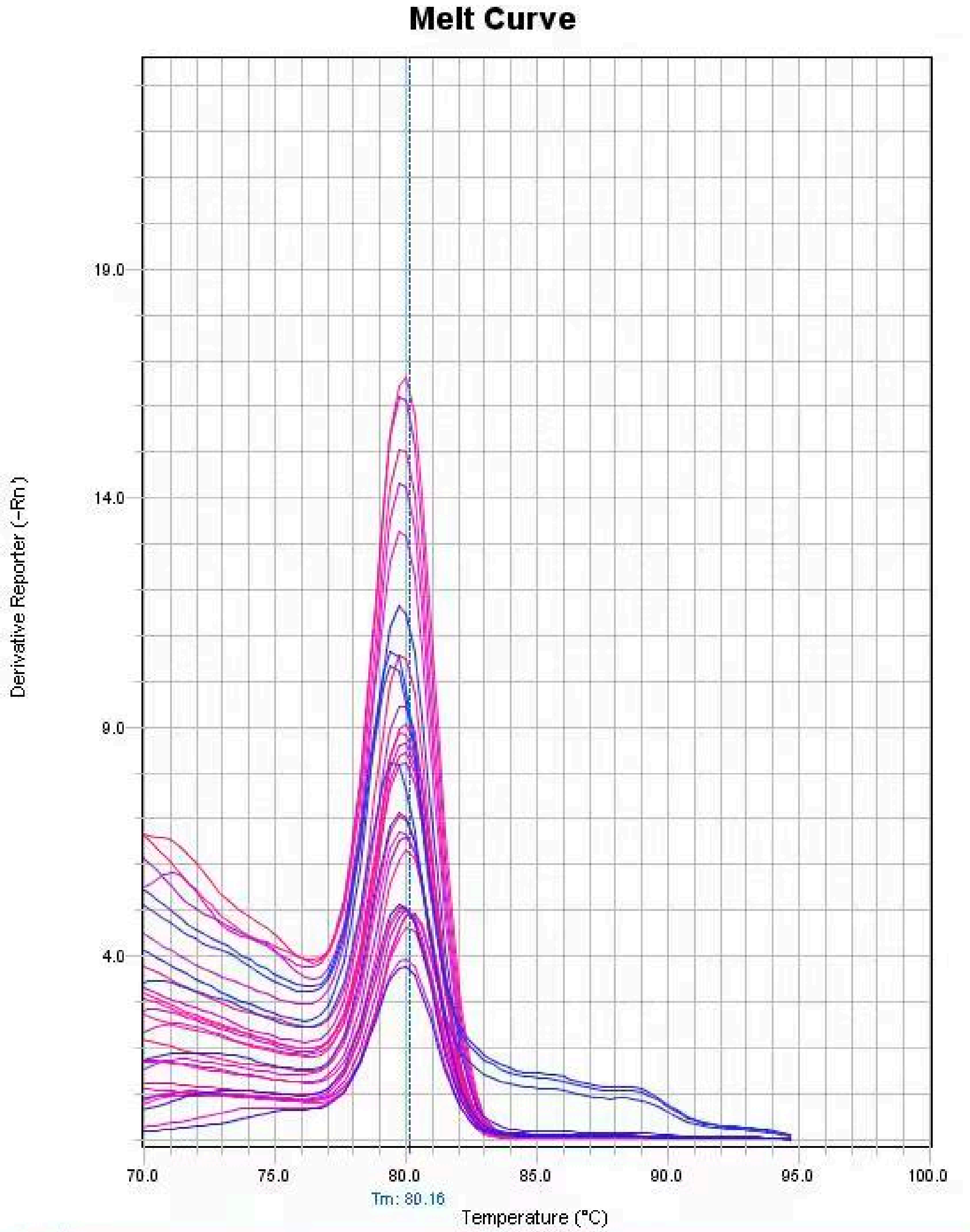

Figure S2. Conjugative transfer of pR55 from *E. coli* C600 to *S. enterica* H9812. Panel A, growth of transconjugants on *Salmonella*-selective SS plates. Black arrow-indicated black colonies are chloramphenicol-resistant pR55-harboring *S. enterica* H9812. Panel B, PCR confirmation of the presence of pR55 in *S. enterica* H9812 transconjugants. M, DNA size marker; +, positive control; -, negative control; 1-3, different transconjugant colonies.

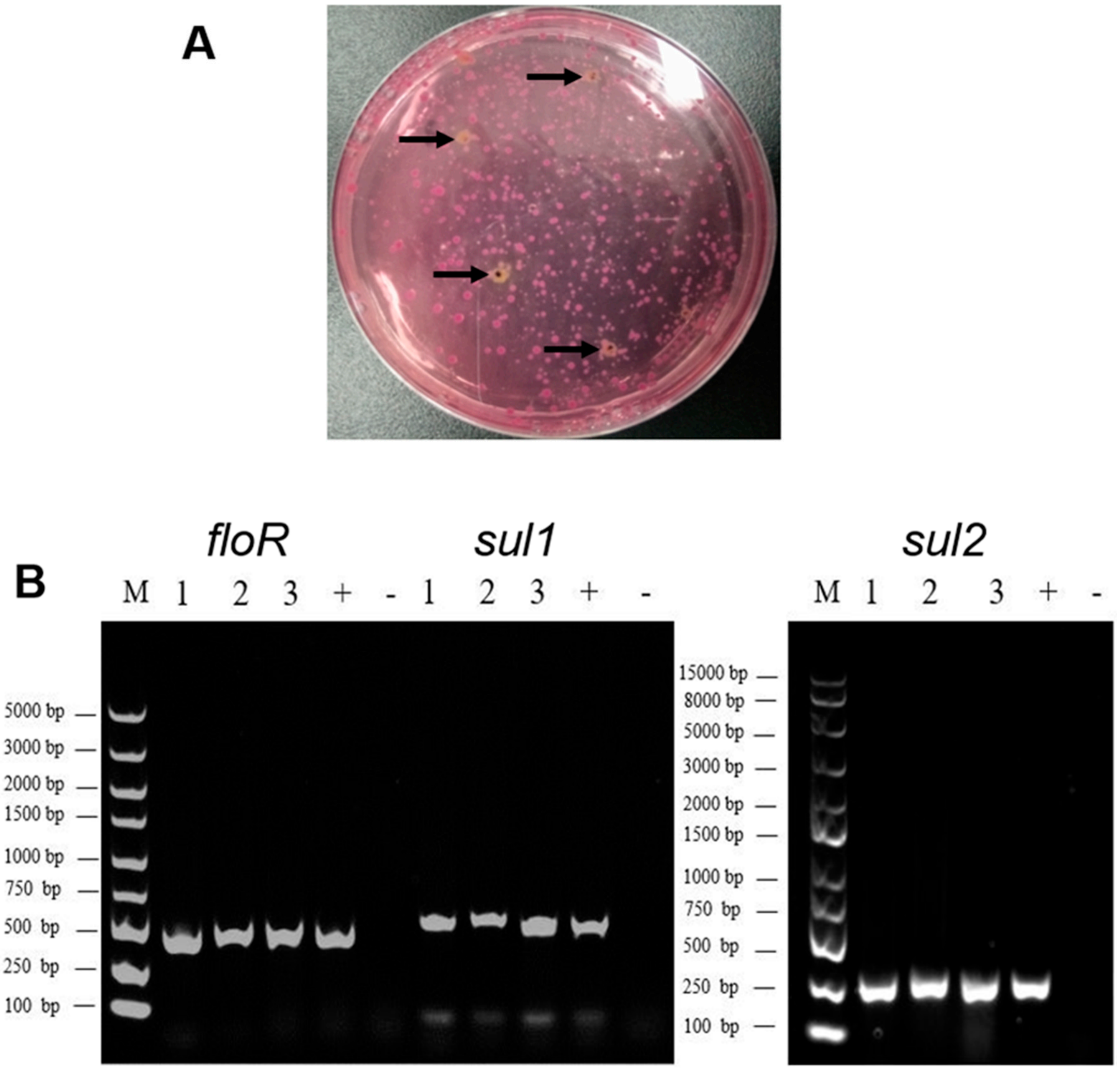

Supplement: Supplementary file 1 [file biology-12-00210-s001.zip › biology-2158430-supplementary.pdf]
